# Antimicrobial effects of nitric oxide in murine models of *Klebsiella* pneumonia

**DOI:** 10.1016/j.redox.2020.101826

**Published:** 2020-12-11

**Authors:** Steffen B. Wiegand, Lisa Traeger, Huan K. Nguyen, Kaitlyn R. Rouillard, Anna Fischbach, Francesco Zadek, Fumito Ichinose, Mark H. Schoenfisch, Ryan W. Carroll, Donald B. Bloch, Warren M. Zapol

**Affiliations:** a Anesthesia Center for Critical Care Research of the Department of Anesthesia, Critical Care and Pain Medicine, Massachusetts General Hospital, Harvard Medical School, 55 Fruit St, Boston, MA, 02114, USA; b Department of Chemistry, University of North Carolina at Chapel Hill, 125 South Rd, Chapel Hill, NC, 27514, USA; c Department of Pediatric Critical Care Medicine, Massachusetts General Hospital, Harvard Medical School, 55 Fruit St, Boston, MA, 02114, USA; d Division of Rheumatology, Allergy and Immunology, Department of Medicine, Massachusetts General Hospital, Harvard Medical School, 55 Fruit St, Boston, MA, 02114, USA

**Keywords:** Lung inflammation, Treatment, Bactericidal, Outcome

## Abstract

**Rationale:**

Inhalation of nitric oxide (NO) exerts selective pulmonary vasodilation. Nitric oxide also has an antimicrobial effect on a broad spectrum of pathogenic viruses, bacteria and fungi.

**Objectives:**

The aim of this study was to investigate the effect of inhaled NO on bacterial burden and disease outcome in a murine model of *Klebsiella* pneumonia.

**Methods:**

Mice were infected with *Klebsiella pneumoniae* and inhaled either air alone, air mixed with constant levels of NO (at 80, 160, or 200 parts per million (ppm)) or air intermittently mixed with high dose NO (300 ppm). Forty-eight hours after airway inoculation, the number of viable bacteria in lung, spleen and blood was determined. The extent of infiltration of the lungs by inflammatory cells and the level of myeloperoxidase activity in the lungs were measured. Atomic force microscopy was used to investigate a possible mechanism by which nitric oxide exerts a bactericidal effect.

**Measurements and main results:**

Compared to control animals infected with *K. pneumoniae* and breathed air alone, intermittent breathing of NO (300 ppm) reduced viable bacterial counts in lung and spleen tissue. Inhaled NO reduced infection-induced lung inflammation and improved overall survival of mice. NO destroyed the cell wall of *K. pneumoniae* and killed multiple-drug resistant *K. pneumoniae in-vitro*.

**Conclusions:**

Intermittent administration of high dose NO may be an effective approach to the treatment of pneumonia caused by *K. pneumoniae.*

## Highlights

• Nitric oxide inhalation reduces viable *Klebsiella pneumoniae* in lung tissue.
• Nitric oxide improves survival rates in murine *Klebsiella* pneumonia.
• Exposure to nitric oxide leads to *Klebsiella* cell wall degradation *in-vitro.*
• Nitric oxide is antibacterial against multiple drug resistant *Klebsiella in-vitro.*

## Graphical abstract

**Figure:**
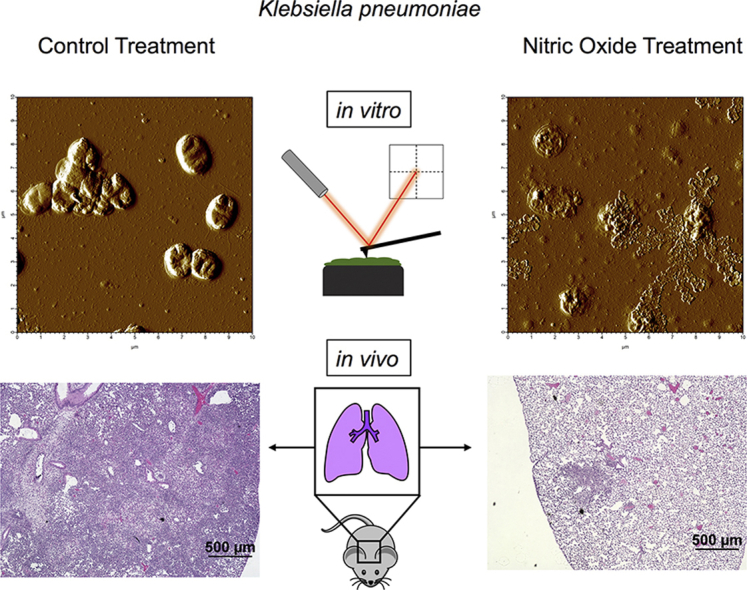

## Abbreviations

CFU: colony forming unit
ESBL: extended-spectrum beta-lactamase
IACUC: Institutional Animal Care and Use Committee
HR: hazard ratio
ICU: intensive care unit
Kp: *Klebsiella pneumoniae*
MDR: multiple-drug, antibiotic resistant
MPO: myeloperoxidase
NIOSH: National Institute for Occupational Safety and Health
NO: nitric oxide
OxyHb: oxygenated hemoglobin
ppm: parts per million
RNS: reactive nitrogen species
SARS-CoV-1: severe acute respiratory syndrome coronavirus 1
SARS-CoV-2: severe acute respiratory syndrome coronavirus 2
SEM: standard error of the mean

## Introduction

Nitric oxide (NO) plays a pivotal role in the regulation of vascular tone, neurotransmission, acute and chronic inflammation, and host defense [1,2]. Low dose NO (up to 80 ppm) is a selective pulmonary vasodilator, approved by the FDA in December 1999 which has been used in over 300,000 babies with persistent pulmonary hypertension of the newborn and for adults to treat pulmonary hypertension and right ventricular failure [[3], [4], [5], [6]].

NO has an antimicrobial effect against a broad range of pathogenic organisms, including viruses (such as SARS-CoV-1, SARS-CoV-2), bacteria and fungi [2,7,8]. At low concentrations (<1 μM), NO functions in intercellular signal transduction [9]. At higher concentrations, NO increases the level of reactive nitrogen species (RNS) [10]. Putative mechanisms of the bactericidal effects of NO and RNOS include nitrosation of cysteine thiols (e.g. in bacterial cells [11]), disruption of iron-sulfur clusters in the respiratory chain of bacteria [12], inhibition of ribonucleotide reductase, an enzyme needed for repairing bacterial DNA [13], and downregulation of ferroportin, the exporter of cellular iron, which is needed for bacterial metabolism [14]. Additional indirect anti-microbial effects of NO include activation of cells involved in host defense, including T-, B-, NK-cells [15] and macrophages [16]. Inhaled NO in humans produces mild bronchodilation [17], increases ciliary beating, which enhances mucociliary clearance [18], and disperses biofilm [19].

*Klebsiella pneumoniae* (Kp) is a frequent cause of health care-associated infections in the intensive care unit (ICU) and especially in patients requiring mechanical ventilation [20,21]. Kp is a member of the so-called “ESKAPE” group of organisms, which includes six of the most clinically relevant bacteria prone to developing antibiotic resistance: ***E****nterococcus faecium,* ***S****taphylococcus aureus,* ***K****lebsiella pneumoniae,* ***A****cinetobacter baumanii,* ***P****seudomonas aeruginosa and* ***E****nterobacter* spp [22]. Kp can develop extended-spectrum beta-lactamase (ESBL) activity and/or carbapenemase activity, which reduces antibiotic treatment options and worsens overall patient outcomes [21,23]. In this study, we developed a murine model of Kp-induced pneumonia and investigated the effects of high dose inhaled NO on pathophysiology and survival.

## Material and methods

A detailed description of the methods can be found in the supplement.

### Animals

This study was approved by Institutional Animal Care and Use Committee (IACUC) at Massachusetts General Hospital, Boston, MA.

Eleven-week-old male C57BL/6 mice (Charles River Laboratory, Worcester, MA, USA) were housed with free access to food and water and acclimated for 24 h prior to experiments. Mice were randomly assigned to breathe either air without NO (control group) or NO in air (treatment group). For each study arm 8–10 mice were used.

### Statistical analysis

Statistical tests were performed using GraphPad Prism (v.8.3. San Diego, CA, USA). Data are expressed as median (25% percentile; 75% percentile) unless otherwise specified. Normal distribution was tested with Shapiro-Wilk test. Two-tailed Student's t-test or One-Way ANOVA with Dunnett's correction for multiple comparisons was performed to compare parametric data with equal variances. If variances differed significantly, a Brown Forsythe ANOVA with Dunnett's correction was performed. A Mann-Whitney-U test with two tailed p-value or a Kruskal-Wallis test with Dunn's correction was used for non-parametric data. A Chi-square test was used to compare neutrophil infiltration scores. A Log-rank test was performed to detect differences in survival. A p-value less than 0.05 was considered significant (* = p < 0.05; ** = p < 0.01; *** = p < 0.001).

## Results

NO inhalation reduces the number of viable Kp bacteria in lung tissue in a dose- and time-dependent manner.

**Fig. 1:**
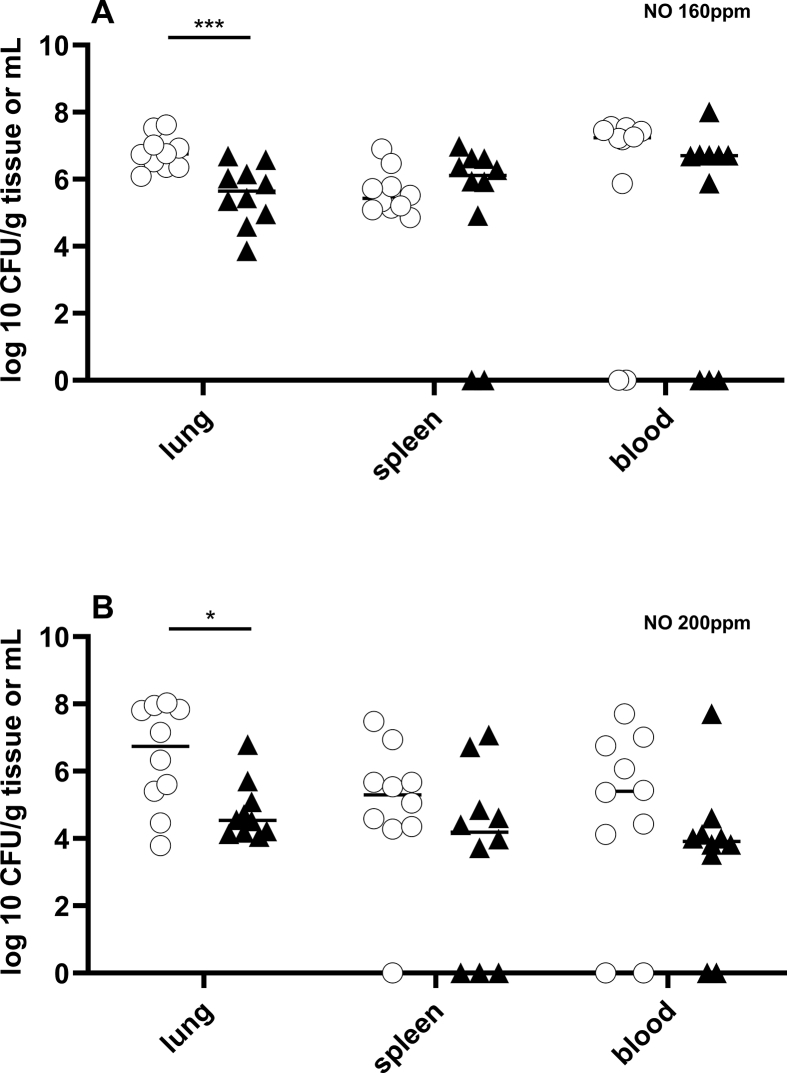
**Breathing NO reduced Kp CFUs in lung tissue in a dose dependent manner.** Mice were infected with 2,000 CFU Kp per animal and treated with NO (160, 200 ppm [black triangle]) or air [white circle], 6 h after inoculation. Mice were sacrificed after 48 h. Inhalation of 160 ppm NO **(A)** or 200 ppm NO **(B)** decreased numbers of bacteria in lungs compared to mice treated with air alone (NO 160 ppm: n = 10, air: n = 10 and NO 200 ppm: n = 10, air: n = 10). NO had no effect on the number of CFUs in the spleen or blood of infected mice at any of the NO concentrations that were tested.

To investigate the effect of NO on Kp-induced pneumonia, mice were inoculated (by inhalation) with Kp (2000 CFUs) and treated by breathing either NO in air or air alone (control) for 48 h. Bacterial CFUs in lung, spleen and blood were measured 48 h after inoculation. Mice treated with 160 or 200 ppm NO had decreased numbers of bacteria in their lungs compared to mice breathing air alone (NO 160 ppm vs air: 5.6 log10 CFU/g (4.9; 6.3) vs. 6.8 log10 CFU/g (6.4; 7.1) p = 0.001; and NO 200 ppm vs air: 4.5 log10 CFU/g (4.2; 5.2) vs. 6.7 log10 CFU/g (5.2; 7.9) p = 0.029) (Fig. 1A and B). Treatment with 80 ppm NO did not have a significant effect on CFUs in lung tissue (NO 80 ppm vs air: 5.8 log10 CFU/g (5.1; 6.7) vs. 6.6 log10 CFU/g (6.3; 7.3), p = 0.102) (Figure S1). NO had no effect on the number of CFUs in the spleen or blood of infected mice at any of the inhaled NO concentrations that were tested.

**Fig. 2:**
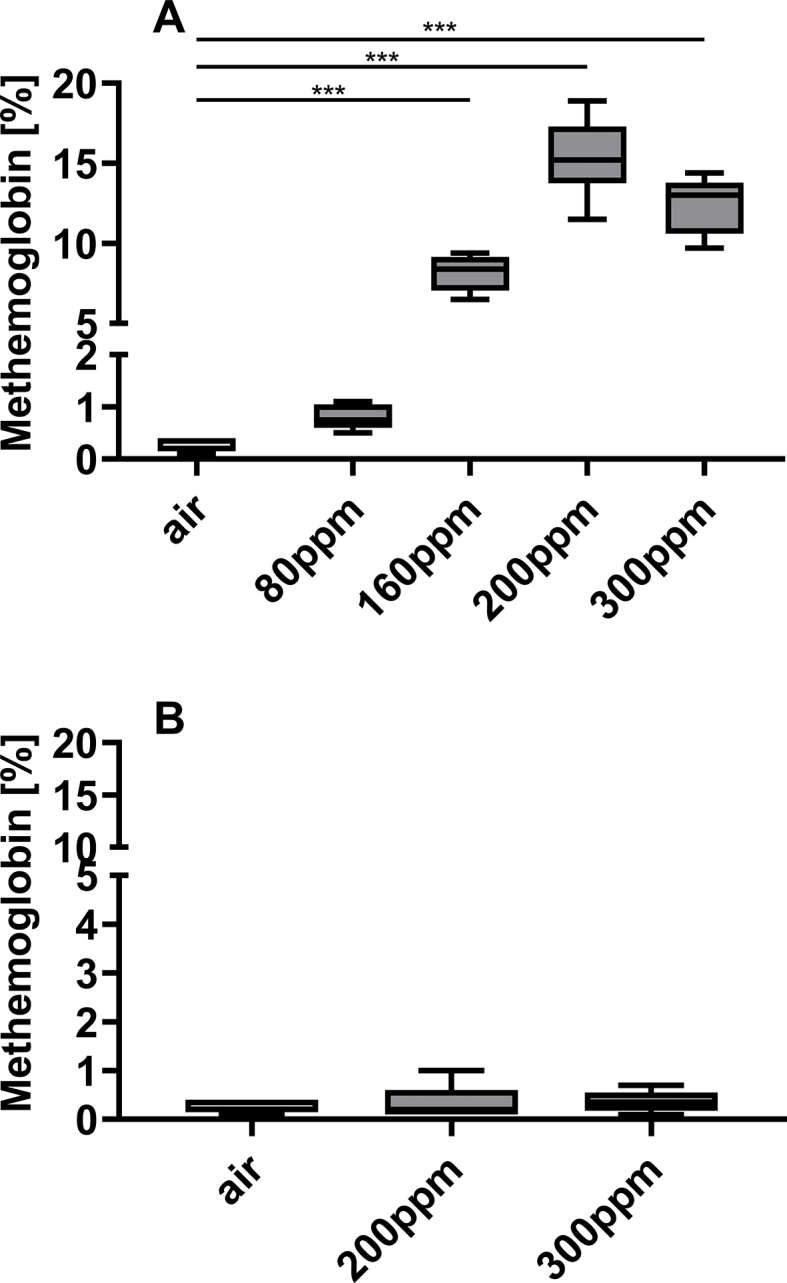
NO inhalation increased methemoglobin levels, which returned to baseline levels 1.5 h after discontinuation of treatment. Mice were infected with 2,000 CFU Kp per animal and treated with NO or air. Mice were sacrificed after 48 h. (A) Methemoglobin levels were increased in most of the NO treated groups compared to air treated controls (NO 80 ppm: n = 8, NO 160 ppm: n = 5, NO 200 ppm: n = 9, NO 300 ppm: n = 5 and air treated controls: n = 9) (*** = p < 0.001). (B) Methemoglobin levels were similar among all groups 1.5 h after stopping NO admission (NO 200 ppm: n = 5, NO 300 ppm: n = 6, air alone: n = 9).

A potential adverse effect of inhaling high concentrations of NO is oxidation of the heme group of hemoglobin, producing methemoglobin. Methemoglobin does not carry oxygen, and increased methemoglobin levels limit the concentration of inhaled NO and the duration of treatment. The enzyme cytochrome *b*5 reductase, also known as methemoglobin reductase, catalyzes the reaction of MetHb to oxygenated hemoglobin (OxyHb) in red blood cells. To determine whether breathing high NO concentrations in air are associated with elevated levels of methemoglobinemia, we measured methemoglobin concentrations in blood after treatment with NO (Fig. 2A). Mice continuously breathing 200 ppm NO for 24 h had median methemoglobin levels of 15.2% (13.8; 17.3) when measured immediately after treatment (Fig. 2A). Administration of 300 ppm NO for 12min or 30min produced a median methemoglobin level of 13% (10.6; 13.8) and 41.3% (40.8; 51.7) (measured directly after treatment), respectively, suggesting that treating with NO at high concentration for the shorter duration might be safer (Fig. 2A). For mice treated with 200 ppm NO for 24 h or 300 ppm NO for 12 min, methemoglobin levels returned to baseline (less than 1%) 1.5 h after discontinuation of NO treatment (Fig. 2B) indicating normal MetHb reductase activity. Furthermore, in mice receiving 200 ppm for 24 h, the reduction of MetHb to OxyHb by MetHb reductase compensates the continuous administration of NO. Treatment with 300 ppm NO for longer durations were not safe for mice and increased MetHb levels to life-threatening levels. The MetHb reductase was not able to compensate MetHb production by NO.

**Fig. 3:**
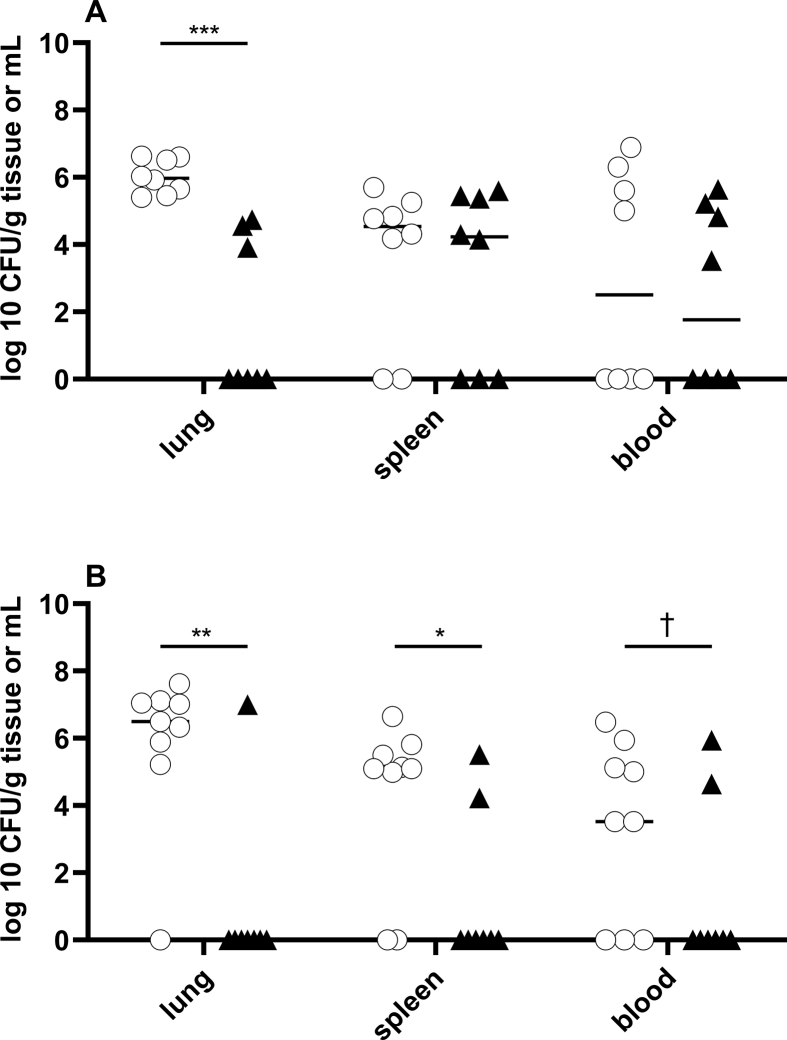
Breathing high dose NO reduced Kp CFUs in lung tissue, and starting treatment with NO early reduced CFUs in both lung and spleen. Mice were infected with 2,000 CFU Kp per animal and treated with intermittent NO (300 ppm) [black triangle] or air [white circle]. Mice were sacrificed after 48 h. **(A)** Intermittent treatment with 300 ppm NO, starting 6 h after inoculation, reduced bacterial CFUs in lung tissue compared to controls (p < 0.001), but not in spleen (p = 0.795) or blood (p = 0.6) (NO 300 ppm: n = 8, air: n = 8) (*** = p < 0.001) **(B)** Early treatment, initiated immediately after inoculation, reduced CFUs in lung and splenic tissue, but not in blood (NO 300 ppm: n = 8, air: n = 9) (* = p < 0.05; ** = p < 0.01). There was a trend towards reduction in bacteremia (bacteremia: air: 6/9 vs. NO 300 ppm: 2/8 (chi-square test: † = p = 0.086).

Because treatment with NO 300 ppm for 12min did not produce excessive methemoglobin levels, and because the level of methemoglobin returned to baseline 1.5 h after ending NO inhalation, we tested the effect of a regimen of intermittent 300 ppm NO on bacterial CFUs in mice infected with Kp. Six hours after inoculation, mice were treated with 300 ppm NO for 12min every 3 h for 48 h. Compared to control mice breathing air alone, intermittent treatment with NO reduced bacterial CFU/g in lung tissue (6 log10 CFU/g (5.5; 6.6) vs. 0 log10 CFU/g (0; 4.4) p < 0.001) (Fig. 3A). However, intermittent, high dose NO had no effect on the number of CFUs in either the spleen or blood of infected mice (Fig. 3A).

To investigate the potential effect of early treatment with NO on Kp-infection, mice were treated with intermittent NO (300 ppm) or air alone, immediately after tracheal inoculation. As was observed in mice treated with intermittent NO 6 h after infection, early NO inhalation was effective in eliminating bacterial CFUs from the lungs (intermittent NO 300 ppm vs air: 0 log10 CFU/g (0; 0) vs. 6.5 log10 CFU/g (6.5 log10 CFU/g (5.6; 7.1), p = 0.004) (Fig. 3B). In contrast to mice treated 6 h after infection, early treatment with intermittent NO at 300 ppm also reduced bacterial CFUs in splenic tissue (NO vs air alone: 0 log10 CFU/g (0; 3.2) vs. 5.1 log10 CFU/g (2.5; 5.7), p = 0.034) (Fig. 3B). In addition, there was a trend towards reduction of bacteremia, with bacteremia occurring in 6 of 9 mice treated with air alone compared to 2 of 8 mice treated by breathing high dose, intermittent NO (chi-square test: p = 0.086).

Taken together, the results show a dose dependent effect of NO on decreasing bacterial CFUs in the lung tissue of mice infected with Kp. Intermittent administration of NO allowed a higher concentration of NO to be used, without causing excessive methemoglobinemia, and was effective in reducing bacterial CFUs in the lung. Early treatment (immediately after bacterial inoculation) with high dose NO, eliminated viable bacteria in the lung and decreased the bacterial CFUs in the spleen.

### NO treatment reduces Kp*-*induced neutrophil infiltration

**Fig. 4:**
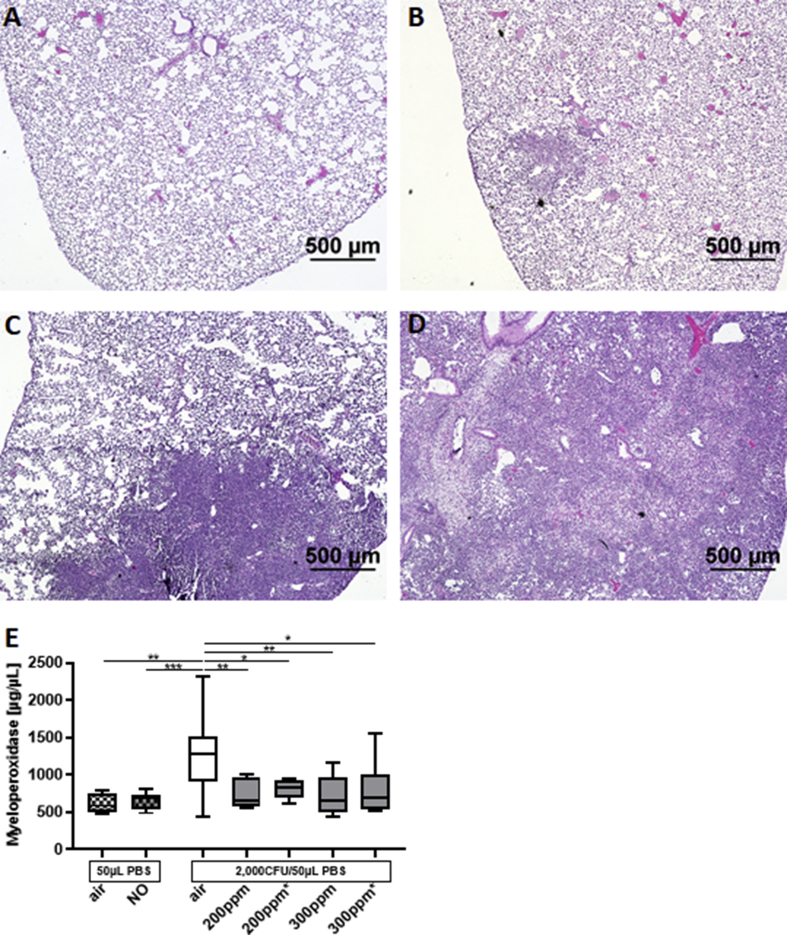
**Treatment with NO decreased lung inflammation and MPO levels in lung extracts.** Mice were inoculated with 2000 CFU of Kp and received NO 200 ppm (continuously) or 300 ppm (intermittently) or air alone for 48 h. **(A**–**D)** Lung sections were fixed and stained with H&E and examined under light microscopy. Representative sections showing **(A)** no infiltration of neutrophils and **(B)** mild infiltration (Score:1), **(C)** intermediate infiltration (Score:2) and **(D)** widespread infiltration (Score:4). No section in this collection of tissues had an inflammation score of 3. (NO 200 ppm: n = 10, NO 300 ppm: n = 10, air: n = 15) **(E)** Myeloperoxidase activity in mice receiving 200 or 300 ppm nitric oxide 6 h after inoculation or immediately after inoculation (^#^) were significantly reduced compared to control mice ((NO 200 ppm+6 h: n = 9, NO 300 ppm+6 h: n = 8, NO 200 ppm^#^: n = 8, NO300 ppm^#^: n = 8, air: n = 37 (* = p < 0.05; ** = p < 0.01; (*** = p < 0.001). There was no difference among the different NO groups.

Inoculation with Kp causes pneumonia in mice and leads to lung damage consisting of alveolar edema, infiltration of neutrophils and abscess formation. Histopathology was used to investigate the effect of NO on Kp-induced neutrophil infiltration of the lung. A semi-quantitative grading system was used to estimate the extent of lung neutrophil infiltration, ranging from 1 (no or few inflammatory cells) to 4 (nearly all portions of the lung containing inflammatory cells) (Fig. 4A–D). Mice received continuous NO (200 ppm) or intermittent NO (300 ppm for 12min every 3 h) or air. More than 50% (8/15) of the air breathing mice had a lung inflammation score of ≥2 with a mean of 1.7 ± 0.2 (SEM). Mice treated with either continuous NO (200 ppm) or intermittent NO (300 ppm) had only mild or no infiltration of neutrophils, with a lung inflammation score of 1.0±0.0. The proportion of mice breathing air alone that had an inflammation score ≥2 was 53% (8/15); none of the 20 NO-treated mice had an infiltration score greater than 1 (Chi-square test: p < 0.001).

Myeloperoxidase (MPO) is an enzyme that is highly expressed in neutrophils and produces hypohalous acids in response to bacterial infection [24]. The activity of MPO was measured in the homogenates of lungs obtained from mice 48 h after inoculation with 2000 CFU Kp. Mice breathed air alone or air with continuous NO (200 ppm) or air with intermittent NO (300 ppm for 12min every 3 h). In mice treated with air alone, infection with Kp increased MPO activity (Fig. 4E) (infected vs uninfected mice; 1282 μg/μL (907; 1510) vs. 587 μg/μL (495; 753); p = 0.008). In infected mice, NO treatment (200 ppm continuously or 300 ppm intermittently) reduced MPO activity compared to air breathing controls (NO 200 ppm vs air: 797 μg/μL (628; 940) vs. 1282 μg/μL μg/μL (907; 1510), p = 0.006. NO 300 ppm vs air: 660 μg/μL (545; 969) vs. 1282 μg/μL (907; 1510) p = 0.005) (Fig. 4E). There was no difference in MPO activity between the two NO regimens (200 ppm or 300 ppm). In addition, the timing of NO treatment, whether immediately after inoculation or 6 h after inoculation, did not affect MPO activity (Fig. 4E). Taken together, the histopathology and MPO activity results show that NO treatment reduces lung inflammation in mice infected with Kp*.*

### High dose, intermittent NO administration reduced overall mortality

**Fig. 5:**
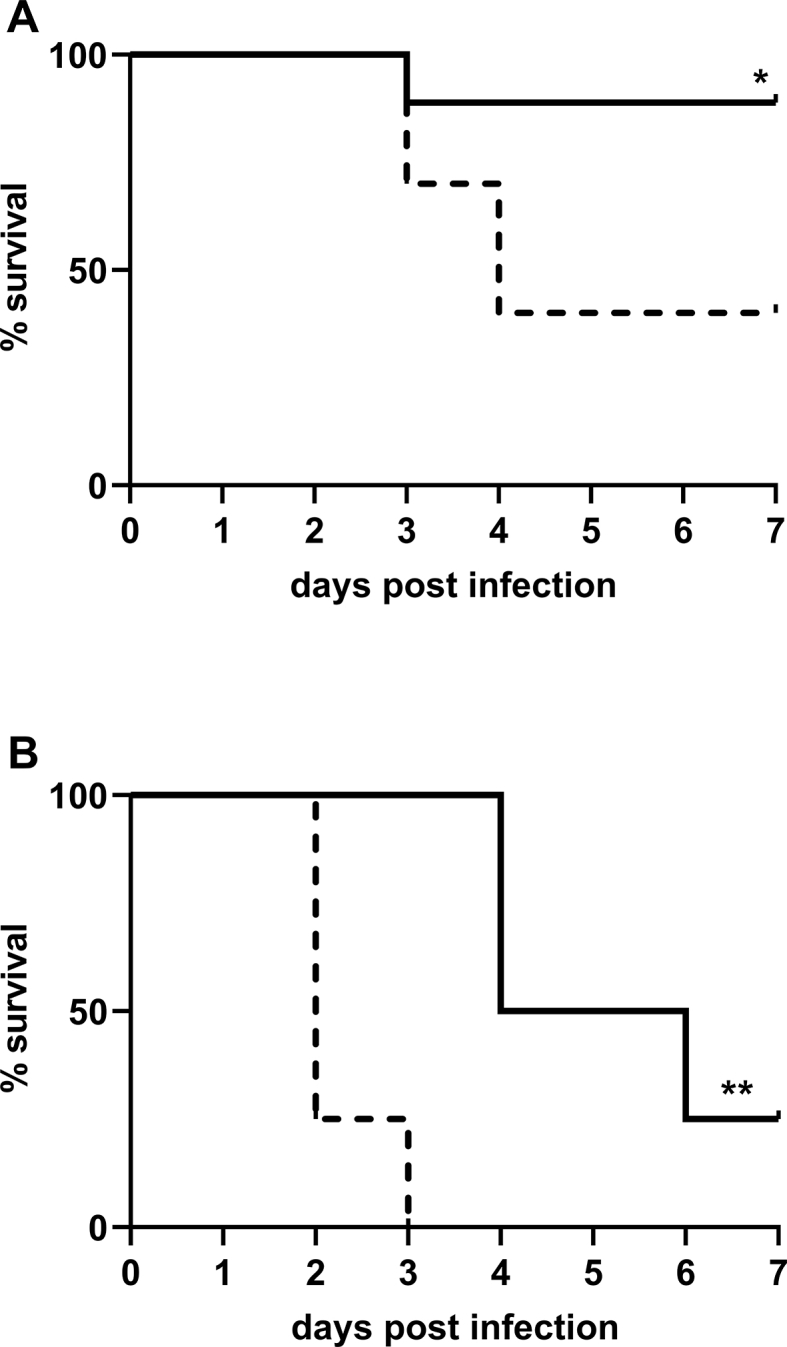
**High dose intermittent NO treatment improved overall survival compared to air breathing controls.** Mice were inoculated with **(A)** 2000 CFU or **(B)** 3500 CFU and received either 12min intermittent treatment with 300 ppm NO every 3 h for 48 h [full line] or air [dashed line]. Overall survival was increased in mice receiving treatment with NO compared to mice in control group (**(A)** Air breathing mice: n = 10, intermittent NO-treated-mice: n = 9 (* = p < 0.05); **(B)** Air breathing mice: n = 4, intermittent NO-treated-mice: n = 4) (** = p < 0.01).

In mice infected with Kp, treatment with intermittent high concentration NO (immediately after infection) reduced the number of bacterial CFUs in the lung and spleen. A 7-day survival study was performed to determine whether the observed decrease in bacterial CFUs was associated with improved survival. Mice were inoculated with Kp and treated with air or with intermittent high concentration NO (300 ppm) in air for 12min every 3 h for a total of 48 h, beginning immediately after inoculation. Compared to mice breathing air without NO, a greater percentage of NO-treated mice survived for 7 days (40% vs 89%, p = 0.04) (Fig. 5A). The median survival duration for mice breathing air was 4 days, whereas 8 out of 9 mice treated with NO survived until the end of the study. The Hazard Ratio (HR) for mice breathing air alone was 6.0 (CI 1.36, 26.32).

To further investigate the effect of NO on survival in this murine model, mice were tracheal inoculated with a larger number of bacteria (3500 CFU). Mice treated with intermittent NO (300 ppm) had a median survival of 5 days compared to a median survival of 2 days for air breathing mice (HR: 4.2, p = 0.006). None of the mice in the air breathing group survived longer than 3 days (Fig. 5B). The results show that intermittent administration of 300 ppm NO improved survival in this murine model of Kp-induced pneumonia.

### Nitric oxide causes cell wall destruction in Kp

**Fig. 6:**
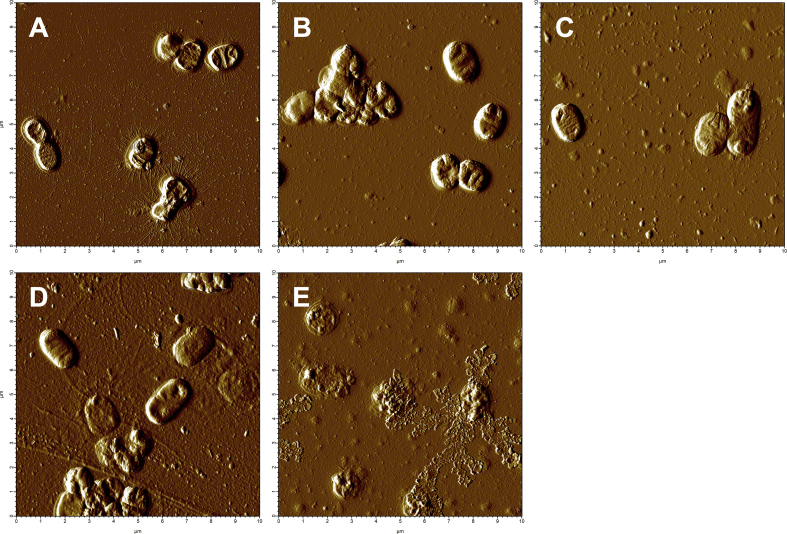
**NO from an NO donor compound (spermine NONOate), led to degradation of Kp cell wall *in-vitro*. (A)** Kp exposed to **(A)** PBS or **(B)** spermine, which does not release NO, showed a smooth intact cell surface. **(C)** NO 0.1 μmol and **(D)** 1 μmol had no effect on the appearance of Kp cell wall. **(E)** NO 10 μmol led to degradation of the cell wall of Kp and spillage of organelles.

To investigate whether NO damages the bacterial cell wall of Kp, we used atomic force microscopy to examine the effect of NO released from the nitric oxide donor spermine NONOate (0.1 μmol–10 μmol) on the bacterial cell wall. Kp that were not exposed to spermine NONOate (PBS or spermine) had a smooth, intact surface without visible damage and had a thickness (Z-height) between 300 and 500 nm (Fig. 6A and B and figure S2A). NO exposure (0.1 μmol or 1 μmol) did not alter the integrity of the bacterial cell wall or bacterial Z-height (Fig. 6C, D and figure S2B, C). Exposure to a higher concentration of NO (10 μmol) damaged the bacterial cell wall, resulting in extrusion of cell organelles and causing an average 60–80% reduction in Z-height (Fig. 6E and figure S2D). The results suggest that NO released by an NO donor compound has a direct dose-dependent bactericidal effect on Kp, mediated by bacterial cell wall degradation.

NO released from a nitric oxide donor is effective against multi-resistant Kp*.*

**Fig. 7:**
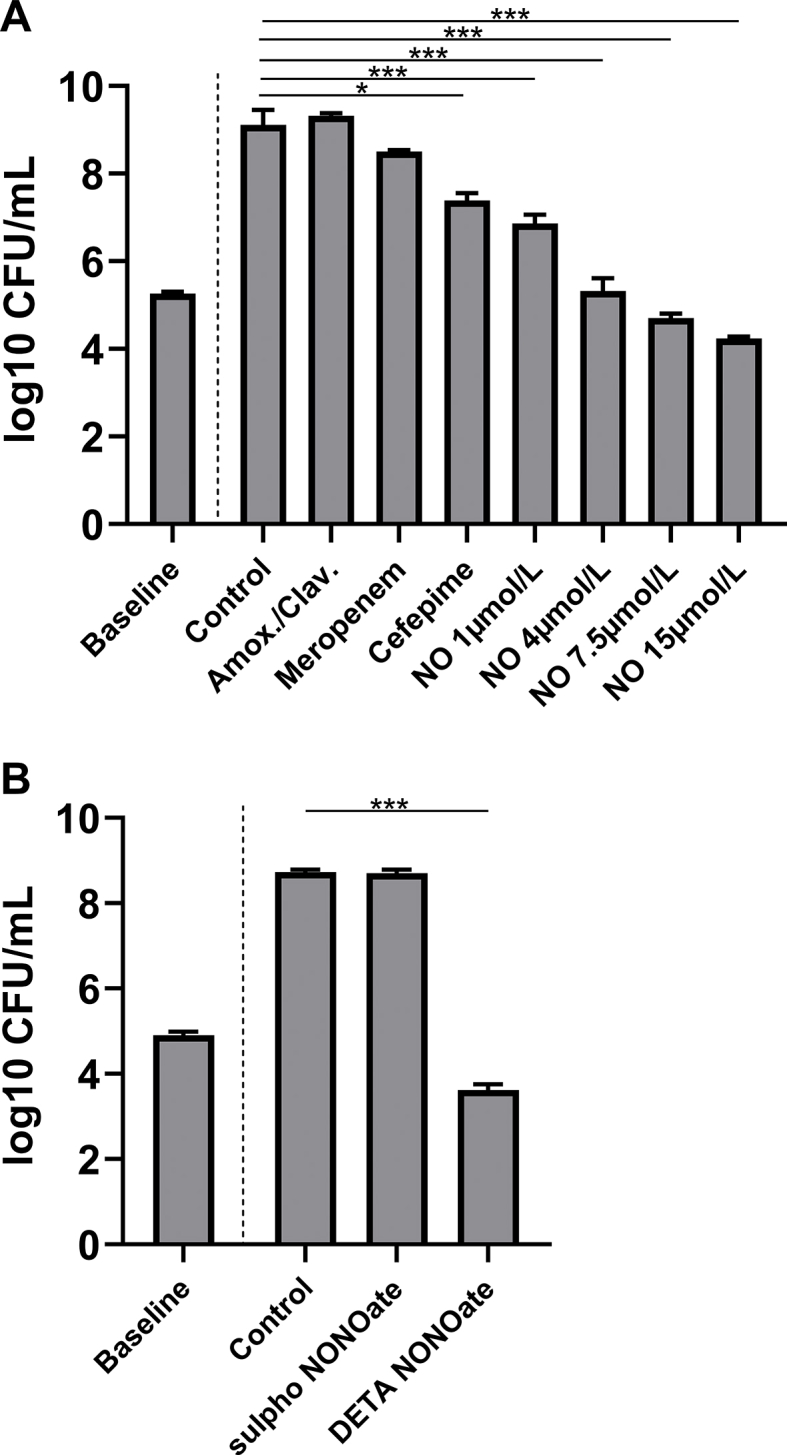
**Exposure to NO released from an NO donor (DETA NONOate) reduced growth of multiple drug resistant Kp in a dose-dependent manner *in-vitro*. (A)** After a 6 h incubation period, bacterial CFUs of multiple-drug resistant Kp increased and addition of amoxicillin/clavulanic acid 32/16 μg/mL or meropenem 2 μg/mL had no effect on the number of CFUs compared to bacteria grown in control media. Cefepime 8 μg/mL has a small effect on Kp CFUs (7.4 log10 CFU/mL (7.2; 7.6), vs. 9.1 log10 CFU/mL (9.0; 9.5), * = p < 0.05) compared to media alone. Exposure with NO 1 μmol/L (6.9 log10 CFU/mL (6.8; 7.1)); 4 μmol/L (5.3 log10 CFU/mL (5.1; 5.6)); 7.5 μmol/L (4.7 log10 CFU/mL (4.6; 4.8)); and 15 μmol/L (4.2 log10 CFU/mL (4.1; 4.3)) reduced Kp CFUs compared to media alone (9.1 log10 CFU/mL (9.0; 9.5)) (each group: n = 10, except controls: n = 20). NO 7.5 μmol/L and 15 μmol/L were bactericidal.; (*** = p < 0.001). **(B)** Exposure with sulpho NONOate at 2.5 mg/mL, which releases nitrous oxide instead of nitric oxide, had no effect on CFUs compared to bacteria grown in media (8.7 log10 CFU/mL (8.7: 8.8) vs. 8.7 log10 CFU/mL (8.7; 8.8); p = 0.96), but 2.5 mg/mL of DETA NONOate, which corresponds to 15 μmol/L NO, was bactericidal (*** = p < 0.001) (each group: n = 5).

An *in-vitro* study was performed to investigate the effect of NO on multiple-drug, antibiotic resistant (MDR) Kp. The Kp strain used in these studies had known resistance to amoxicillin/clavulanic acid, meropenem, and cefepime, according to antibiotic sensitivity data from the vendor and verified by our institutional microbiology laboratory. MDR Kp suspensions were incubated for 6 h with NO from an NO donor (ranging from 1 μmol/L to 15 μmol/L [DETA NONOate: 0.167 mg/mL to 2.5 mg/mL]), sulpho NONOate, which releases nitrous oxide, but not NO (2.5 mg/mL) or media alone, and CFUs were determined. Compared to MDR Kp grown in media alone, the addition of amoxicillin/clavulanic acid (32/16 μg/mL) had no effect on bacterial CFUs, nor did the addition of meropenem (2 μg/mL). Cefepime (8 μg/mL) has a small (but significant) effect on CFUs compared to controls (Fig. 7A). The addition of NONOate to MDR Kp bacterial suspension caused a dose-dependent reduction in CFUs/mL. (Fig. 7A). NO at 7.5 μmol/L was bactericidal, reducing CFUs by the end of the incubation period to lower CFU levels prior to the incubation period. The addition of sulpho NONOate (2.5 mg/mL) did not inhibit growth of Kp compared to media alone (Fig. 7B). The results show that NO released by an NO donor inhibits growth of MDR Kp in a dose-dependent manner.

## Discussion

The objectives of this study were to investigate the effect of breathing high dose NO on bacterial burden and disease outcome in murine *Klebsiella* pneumonia, and to begin to elucidate the mechanisms by which exposure to NO affects Kp *in-vitro*. Dose response experiments showed that with increasing concentration of inhaled NO, Kp CFUs in lung tissue were reduced compared to controls. Increasing the NO concentration was limited, however by circulating blood methemoglobin production. Shorter treatment (12min) with high dose NO (300 ppm) led to safer methemoglobin levels, which returned to baseline levels 1.5 h after discontinuation of inhalation. Using this shorter treatment regimen, starting 6 h after inoculation, breathing NO in air was effective in reducing CFUs in lung tissue but not in spleen or blood. If treatment with intermittent high dose NO was started immediately after inoculation, Kp CFUs in both lung and spleen were reduced. In addition, NO reduced lung inflammation in mice infected with Kp, as determined by histology and by measuring the MPO activity of lung extracts. High dose intermittent NO improved survival in this murine model of Kp-induced pneumonia. *In-vitro* exposure of Kp to an NO donor showed a bactericidal effect, which was assessed using atomic force microscopy. An NO donor decreased the growth of a multiple drug resistant Kp strain.

In this model of Kp-induced pneumonia, breathing higher NO concentrations (160 ppm, 200 ppm) was effective in reducing bacterial CFUs in lung tissue compared to air breathing controls. Lower inhaled NO concentrations (80 ppm), however, were ineffective for the treatment of Kp-derived pneumonia. These results are in contrast to those of Jean and Webert, who reported that low concentrations of NO (10 and 40 ppm) were able to reduce CFUs of *P. aeruginosa* in rodent lung tissue [25,26]. The relative insensitivity of Kp to low inhaled NO concentrations may be the result of organism-dependent differences. Workman et al. suggested that species such as Kp require exposure to higher concentrations of NO to achieve complete bacteriostasis [27]. In this study, administration of high dose NO was achieved by limiting the duration of treatment with high dose NO to 12 min every 3 h, thereby avoiding high levels of methemoglobinemia.

Treatment of mice with high dose intermittent NO, starting 6 h after infection, decreased the number of CFUs in the lungs, but not the spleen. In contrast, initiating treatment immediately after infection decreased the number of CFUs in both the lung and spleen. The increased efficacy of early treatment may be of clinical importance for patients who experience an observed aspiration event or are at increased risk of aspiration because of intubation, stroke or dysphagia [28].

The beneficial effect of NO inhalation on Kp CFUs in lung was associated with the absence of neutrophil infiltration in lung tissue and decreased MPO activity in lung extract. NO may exert a direct effect on inflammation, by decreasing the bacterial burden. NO may also produce an indirect effect on inflammation: previous investigators showed that NO inhibits the expression of many genes believed to be involved in inflammatory cascades, including nuclear factor-κB [29], tumor necrosis factor-α [30], and interleukin-1 [30]. In pigs challenged with intravenous lipopolysaccharide infusion, NO inhalation up-regulates glucocorticoid receptor expression, which is known to mediate anti-inflammatory responses [31].

To investigate the underlying mechanism as to how NO exerts its bactericidal effect on Kp, we exposed Kp *in-vitro* to NO released from NO donor compounds and found that NO damages the bacterial cell wall of Kp in a dose dependent manner. Deupree and colleagues observed a similar morphological effect of NO on the bacterial cell wall of *P. aeruginosa* and *E. coli* [32]*.* Degradation of the Kp cell wall, potentially mediated by nitrosation [11], would be expected to increase cell wall permeability and may therefore facilitate entry of antibiotics into the organisms.

High dose NO had anti-microbial effects on both antibiotic sensitive and antibiotic resistant Kp *in-vitro*. Treatment with intermittent high dose NO may be especially beneficial in patients who develop frequent or chronic infections with antibiotic resistant organisms. Such patients include those with cystic fibrosis or chronic obstructive pulmonary disease. A phase I study involving eight cystic fibrosis patients with chronic pulmonary infections, intermittently breathing NO (160 ppm in air), has already been completed and was shown to be safe [33].

A potential limitation of the use of high concentrations of NO to treat infection is the adverse effects of increased levels of inhaled nitrogen dioxide (NO_2_). Nitrogen dioxide reacts with water to form nitrous and nitric acid and can thereby damage epithelial cells [34]. In this study, administration of NO 300 ppm to mice produced an inhaled NO_2_ level that remained below 2 ppm. The National Institute for Occupational Safety and Health's (NIOSH) recommended NO_2_ exposure limit is 1 ppm over a 15-min short-term interval, and the American Conference of Governmental Industrial Hygienists has an threshold limit value of 3 ppm for 8 h [35]. Because the level of NO_2_ increases with FiO_2_, the potential future use of NO to treat infections will require the use of potent NO_2_ scavengers and careful monitoring of NO_2_ levels. A second potential limitation of this study is that the results may not be generalizable to other organisms, including *Enterococcus faecium, Staphylococcus aureus, and Acinetobacter baumanii*, which are prone to develop antibiotic resistance [22]. Future studies should expand high dose nitric oxide treatment to a broad spectrum of bacteria to close this gap in our knowledge.

## Conclusion

In summary, this study demonstrates that intermittent inhalation of 300 ppm NO in air led to reduced Kp CFUs in lung tissue compared to air breathing controls. Early treatment with 300 ppm NO decreased bacterial CFUs in both lung and splenic tissues. The enhanced bacterial clearance could be a result of a direct intrapulmonary bactericidal effect of NO caused by NO-mediated cell wall destruction. Inhalation of NO resulted in a decrease in lung infiltration by neutrophils and an increase in murine survival duration. Future studies should address the potential additive effects of NO in combination with antibiotics for the treatment of respiratory infections.

## Declaration of competing interest

WMZ is on the scientific advisory board of Third Pole Inc., which has licensed patents on electric NO generation. All other authors have nothing to declare.
