# Supplementary material for: Antimicrobial effects of nitric oxide in murine models of Klebsiella pneumonia
Source: Redox Biol. 2020 Dec 11;39:101826. doi: 10.1016/j.redox.2020.101826 (PMC7729265; doi:10.1016/j.redox.2020.101826)
Supplement: Multimedia component 1 [file mmc1.docx]

**Antimicrobial effects of nitric oxide in murine models of *Klebsiella* pneumonia.**

Steffen B. Wiegand, Lisa Traeger, Huan K. Nguyen, Kaitlyn R. Rouillard, Anna Fischbach, Francesco Zadek, Fumito Ichinose, Mark H. Schoenfisch, Ryan W. Carroll, Donald B. Bloch, Warren M. Zapol

**Online Data Supplement**

Preparation of *Klebsiella pneumoniae* suspension

*Klebsiella pneumoniae* (ATCC® 43816™ Manassas, VA, USA) were cultured at 37°C in tryptic soy broth (TSB) overnight (14-18h) with shaking at 225 rotations per minute (rpm). To prepare bacteria in the log phase of growth, 1mL of overnight bacterial culture was added to TSB and incubated for 2.5h. An aliquot of 1mL was centrifuged at 7,500g for 2min and the bacterial pellet was washed three times in sterile phosphate buffered saline (PBS). The bacterial pellet was resuspended in a volume of PBS to achieve an OD_600_, which contained approximately 1x 10^9^ colony forming units (CFU) per mL. Kp suspensions were further diluted with sterile PBS to produce 2,000 +/- 200 CFU/50µL PBS. The number of viable Kp was confirmed by growing triplicates of serial dilutions of bacterial suspension on tryptic soy agar plates.

Induction of pneumonia

Animals were anesthetized with isoflurane 4% with FiO_2_ 1.0. Anesthetized mice were placed in a semi-recumbent position on an intubation stand. After sufficient depth of anesthesia was confirmed and breathing occurred at 2-3 second intervals, mice were intubated with a fiberoptic device (Kent Scientific, Torrington, CT, USA) while constantly receiving isoflurane 1.5-2%. Endotracheal position was confirmed by bilateral chest movement when connecting the endotracheal tube (20G catheter) to a mechanical ventilator (MiniVent Ventilator Model 845, Harvard Apparatus, Holliston, MA, USA) (stroke volume 25µL, ventilation rate 100 breaths/min). A bacterial suspension of Kp (2,000CFU/50µL PBS) was administered with a gel loading pipette tip (ART P100 gel loading pipette tip, Thermo Fisher Scientific, Cambridge, MA, USA) via endotracheal tube. The gel loading pipette was removed and 0.2mL of air was administered via the endotracheal tube to ensure equal distribution of the inoculum. For sham-infected animals, 50µL of sterile PBS was administered intratracheally. If mice did not recover from procedure within 1h and developed signs of imminent death, such as minimal responsiveness to tactile stimulation, mice were excluded and euthanized.

Exposure to inhaled nitric oxide

Mice were randomly assigned to receive either air (FiO_2_: 0.21) or 80, 160, 200, or 300ppm NO (Airgas, Radnar, PA, USA) in air (NO is stored in tanks containing nitrogen. Oxygen was added depending on the NO regimen to get an air-FiO_2_ of 0.21). Six hours after inoculation, mice breathed air or NO with 80,160 or 200ppm in air for 48h. In some experiments, mice were treated with 300ppm NO administered intermittently for 12min every 3h for 48h. To investigate the effect of administering NO shortly after inoculation, mice were treated with either continuous NO (200ppm) in air or intermittent NO (300ppm, in the regimen described above), immediately after inoculation. Plexiglas chambers were used to facilitate NO administration in mouse cages and soda lime was placed inside the chambers to prevent accumulation of CO_2_ and NO_2_. Temperature and humidity were checked hourly during the daytime in the housing area to ensure that environmental conditions during the experimental protocols were identical. For the treatment group NO, NO_2_ and O_2_ levels were measured. NO concentration was measured using a chemiluminescence-based NO analyzer (Sievers NOA-280i, Zysense, Weddington, NC, USA). NO_2_ levels were measured using an NO_2_ analyzer (CAPS NO2 monitor, Aerodyne research, Billerica, MA, USA) and oxygen level were measured using an oxygenmeter (MiniOX3000^®^, Ohio Medical, Gurnee, IL, USA). NO administration to mice using the intermittent 300ppm NO regimen were performed in a modified treatment cage to limit NO_2_ concentrations to less than 2ppm.

Blood and tissue samples

Blood was collected while the mice were under anesthesia with isoflurane (~2.5%) via cardiac puncture, 48h after tracheal inoculation. Each blood sample was divided into two parts to permit bacterial quantification and methemoglobin measurement. Methemoglobin concentrations were measured using a blood gas analyzer (ABL700, Radiometer, Brea, CA, USA).

An incision was made in the inferior *vena cava* and ice-cold PBS was used to flush out circulating blood from the lungs. Lungs were removed *en bloc* from the chest cavity, and the trachea and extraparenchymal airways were removed. The remaining lung tissue was weighed and homogenized in 1mL PBS containing EDTA 1mmol/L with an enclosed, sterile tissue grinder (Fisherbrand, Waltham, MA, USA). Aliquots of lung homogenates were used to quantify bacteria and myeloperoxidase (MPO) activity. To measure the activity of MPO, lung homogenates were centrifuged at 2,000g for 10min and the level of myeloperoxidase activity in the lung extract was determined using a commercially available ELISA (DuoSet 3174, R&D Systems, Minneapolis, MN, USA) according to the manufacturer’s instructions.

Spleens were harvested, weighed and homogenized in the same fashion as the lung tissue. To quantify the bacterial load in lung and spleen homogenates as well as whole blood, homogenates or blood were serially diluted and plated in triplicate on tryptic soy agar to determine Kp CFU. Bacterial CFUs in each tissue were normalized to logarithmic CFU per organ weight or per blood volume, respectively. CFU was recorded as 0 if no bacterial growth was observed.

Histopathology

For histopathology mice were anesthetized and an incision was made in the inferior *vena cava* and ice-cold PBS was used to flush out circulating blood from the lungs. The ventral side of the trachea was surgically exposed and an 18G needle was placed into the trachea and sutured in place. Lungs were inflated with 10% buffered formalin phosphate (Fisher Chemical, NJ, USA) at a pressure of 25cm H_2_O for 25min before the lungs were placed in formalin for 24h and embedded in paraffin. Lung lobe sections (5μm) were stained with haematoxylin and eosin (H&E) and examined under light microscopy (Nikon Eclipse 80i, Nikon Instruments, Melville NY, USA and Retiga 2000R, QIMAGING, Surrey, BC, CA) for evidence of inflammation. Grading of inflammation was determined by scoring neutrophil infiltration as previously described [1] using the following scoring system: 1= few (less than 25% of lung tissue involved) or no inflammatory cells detected; 2= 25-50% of the lung tissue with infiltration by inflammatory cells; 3= inflammatory cells in 50–75% of the lung; 4= almost all lung tissue contained inflammatory infiltrate. Grading of inflammation was performed by two independent investigators blinded to the samples’ origins.

Survival study

To investigate the effect of NO on survival of mice infected with Kp, mice were infected with 2,000CFU/animal and treated with either air alone or intermittent NO in air (300 ppm for 12 min, every 3 hours for 48 hours). If mice developed signs of imminent death, such as minimal responsiveness to tactile stimulation and/or weight loss greater than 20%, mice were euthanized according to guidelines of the local Institutional Animal Care and Use Committee (IACUC) at Massachusetts General Hospital, Boston, MA. All surviving animals were euthanized after 7 days.

Bacterial preparation and atomic force microscopy (AFM)

To investigate an anti-microbial mechanism of NO, we exposed Kp to an NO donor (spermine NONOate) and used AFM to investigate the effect of NO exposure on the cell wall of bacteria. The frozen stock of Kp (ATCC® 43816™ Manassas, VA, USA) was reconstituted in TSB (3mL) at 37°C with shaking overnight. The culture was then diluted in fresh TSB (30mL), grown to 10^8^ CFU/mL, and centrifuged (3,000g for 10min, 25°C). Following centrifugation, the supernatant was discarded, and the remaining pellet was suspended in 20mL of phosphate-buffered saline (PBS) at pH 7.4. For each sample prepared for Atomic Force Microscopy (AFM), freshly cleaved mica (Bruker, Billerica, MA, USA) was incubated first with 250µL of 0.1mg/mL poly-L-lysine (R&D Systems, Minneapolis, MN, USA) for 1h, then with a 250µL aliquot of the Kp suspension in PBS for 1h. Excess liquid was removed with a micropipette after each incubation period. In a 12-well plate, the mica specimen was immersed in a well containing 2mL of spermine (1mg/mL) (Sigma Aldrich, St. Louis, MO, USA) or spermine NONOate (Cayman Chemical Company, Ann Arbor, MI, USA) in different concentrations (0.01mg/mL, 0.1mg/mL, 1mg/mL corresponding to 0.1µmol/L, 1µmol/L, 10µmol/L of NO) in PBS and incubated for 24h at 37°C with shaking. Before immersion, the spermine NONOate solution was adjusted to pH7.4 with HCl. Immediately before AFM imaging, the mica was gently rinsed with Milli-Q water and dried at room temperature.

Tapping-mode AFM images were obtained in air using an MFP-3D AFM system (Asylum Research, Santa Barbara, CA, USA) and AC240TS silicon beam cantilevers (Olympus, Center Valley, PA, USA). Survey scans (40µm x 40µm) were conducted at random locations on the mica substrates before high-resolution images were acquired at 0.25Hz scan rate with a resolution of 1024 x 1024 pixels. To aid visualization of image details, raw images were processed using software provided by Asylum Research.

Nitric oxide sensitivity assay for resistant K. pneumoniae

A suspension of multi-drug resistant Kp bacteria (ATCC®, BAA-2786, strain BK34744) was prepared the same way as described above for antibiotic-sensitive Kp. A baseline bacterial concentration of approximately 10^5^ CFU/mL was used to observe exponential growth. Bacterial suspension was placed in wells of a 96-well-plate. amoxicillin/clavulanic acid 32µg/mL or meropenem 2µg/mL or cefepime 8µg/mL; [Sigma Aldrich, Darmstadt, Germany]) or DETA NONOate (Cayman Chemicals, Ann Arbor, MI, USA) at concentrations ranging from 0.167mg/mL to 2.5mg/mL, corresponding to 1µmol/L to 15µmol/L of NO[2], were added to the samples. Sulpho NONOate (2.5mg/mL) (Cayman Chemicals, Ann Arbor, MI, USA), a NONOate, which releases nitrous oxide, but no nitric oxide, was used as an additional negative control[3,4]. pH levels were adjusted to match baseline pH levels of media (pH7.4). The plate was incubated at 37°C in an incubator with shaking at 300rpm. After 6h, samples were serially diluted, plated in triplicate on tryptic soy agar and incubated. CFUs/mL were calculated after 16h at 37°C.

**References**

[1] M.A. Beck, H.K. Nelson, Q. Shi, P. Van Dael, E.J. Schiffrin, S. Blum, D. Barclay, O.A. Levander, Selenium deficiency increases the pathology of an influenza virus infection, The FASEB Journal. 15 (2001) 1481–1483. https://doi.org/10.1096/fj.00-0721fje.

[2] A.D. Workman, R.M. Carey, M.A. Kohanski, D.W. Kennedy, J.N. Palmer, N.D. Adappa, N.A. Cohen, Relative susceptibility of airway organisms to antimicrobial effects of nitric oxide, International Forum of Allergy & Rhinology. 7 (2017) 770. https://doi.org/10.1002/alr.21966.

[3] C.M. Maragos, D. Morley, D.A. Wink, T.M. Dunams, J.E. Saavedra, A. Hoffman, A.A. Bove, L. Isaac, J.A. Hrabie, L.K. Keefer, Complexes of .NO with nucleophiles as agents for the controlled biological release of nitric oxide. Vasorelaxant effects, J Med Chem. 34 (1991) 3242–3247. https://doi.org/10.1021/jm00115a013.

[4] L.K. Keefer, R.W. Nims, K.M. Davies, D.A. Wink, “NONOates” (1-substituted diazen-1-ium-1,2-diolates) as nitric oxide donors: Convenient nitric oxide dosage forms, in: Nitric Oxide Part A: Sources and Detection of NO; NO Synthase, Academic Press, 1996: pp. 281–293. https://doi.org/10.1016/S0076-6879(96)68030-6.

**
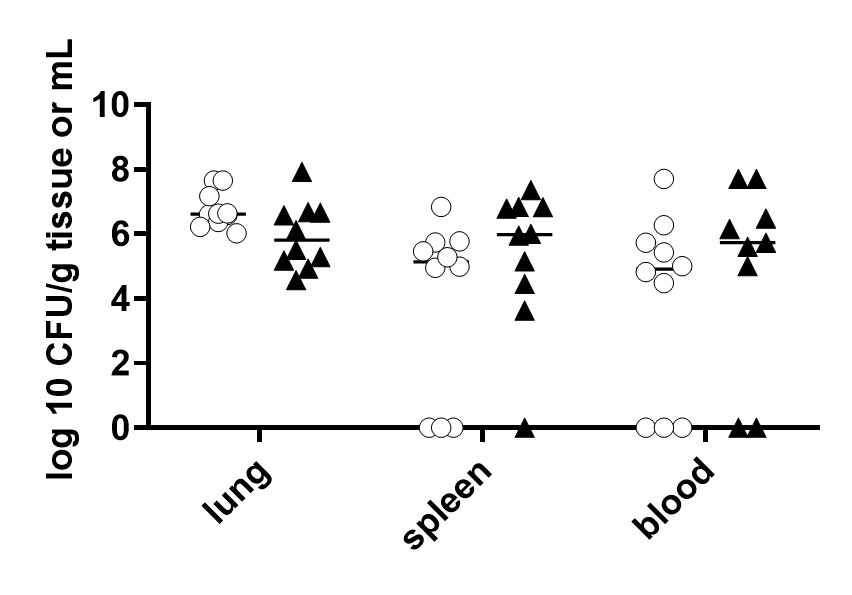
**

**Figure S1. Breathing NO 80ppm had no effect on Kp CFUs.** Mice were infected with 2,000CFU Kp per animal and treated with NO 80ppm [black triangle] or air [white circle], 6h after inoculation. Mice were sacrificed after 48h. CFUs in the lungs, spleen and blood of mice treated with 80 ppm (n=10) were similar to those from mice treated with air (n=10).


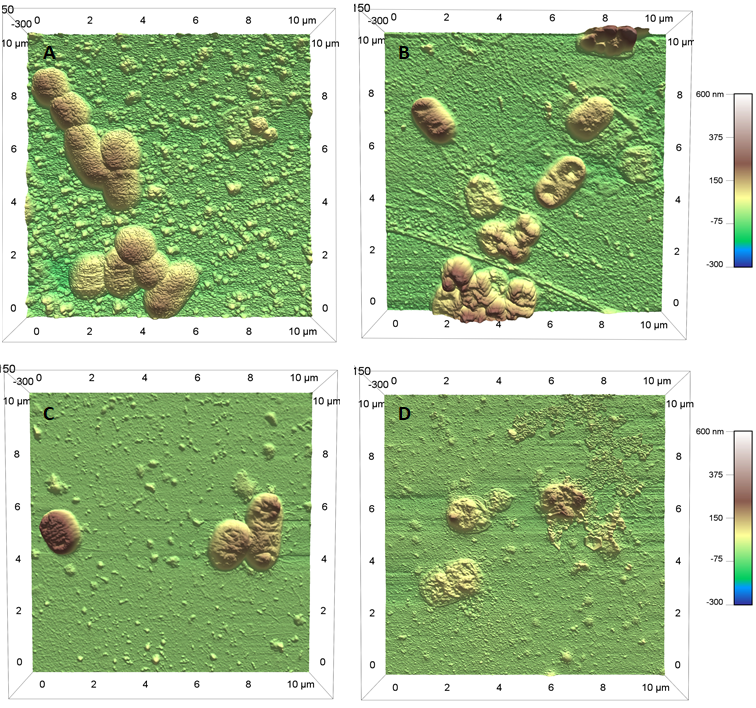


**Figure S2. NO from an NO donor compound (spermine NONOate) led to degradation of Kp cell wall *in-vitro*. (A)** Kp had a Z-height between 300-500nm when treated with PBS only**. (B)** NO 0.1µmol and **(C)** 1µmol had no effect on the appearance of Kp cell wall. **(D)** NO 10µmol led to degradation of the cell wall of Kp, and Z-height was reduced to 100-200nm **(D)**.
